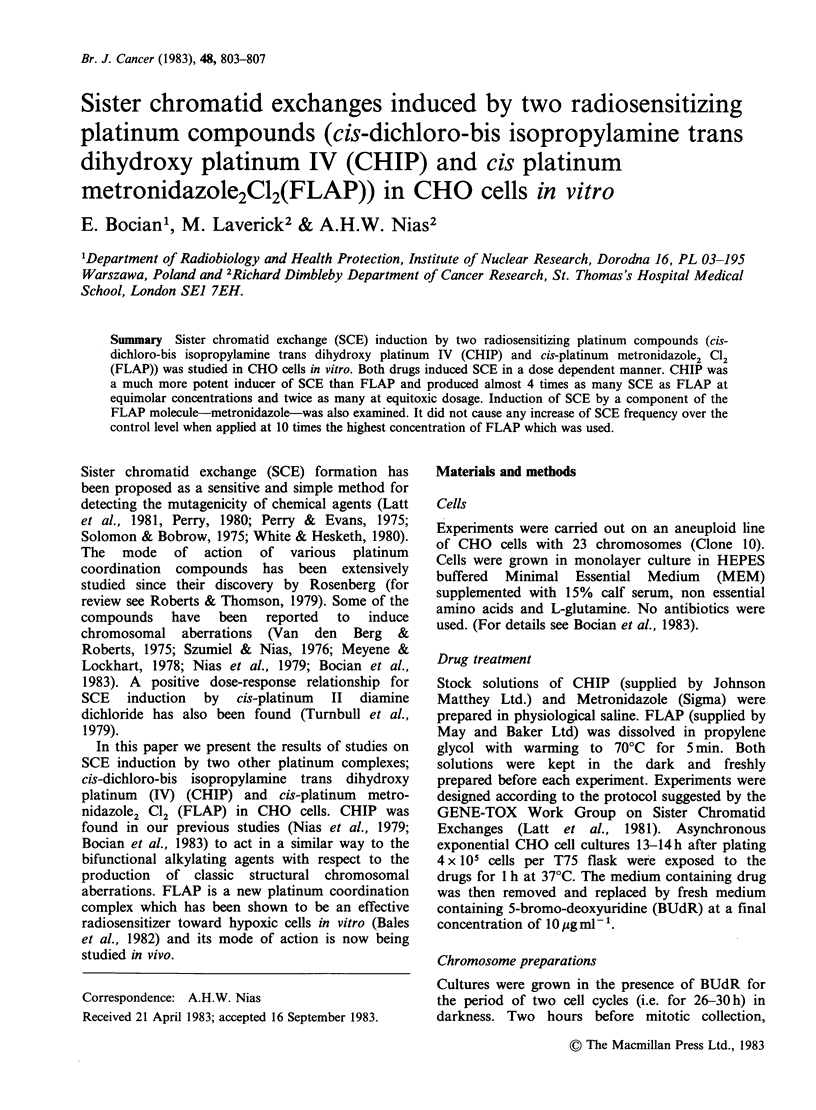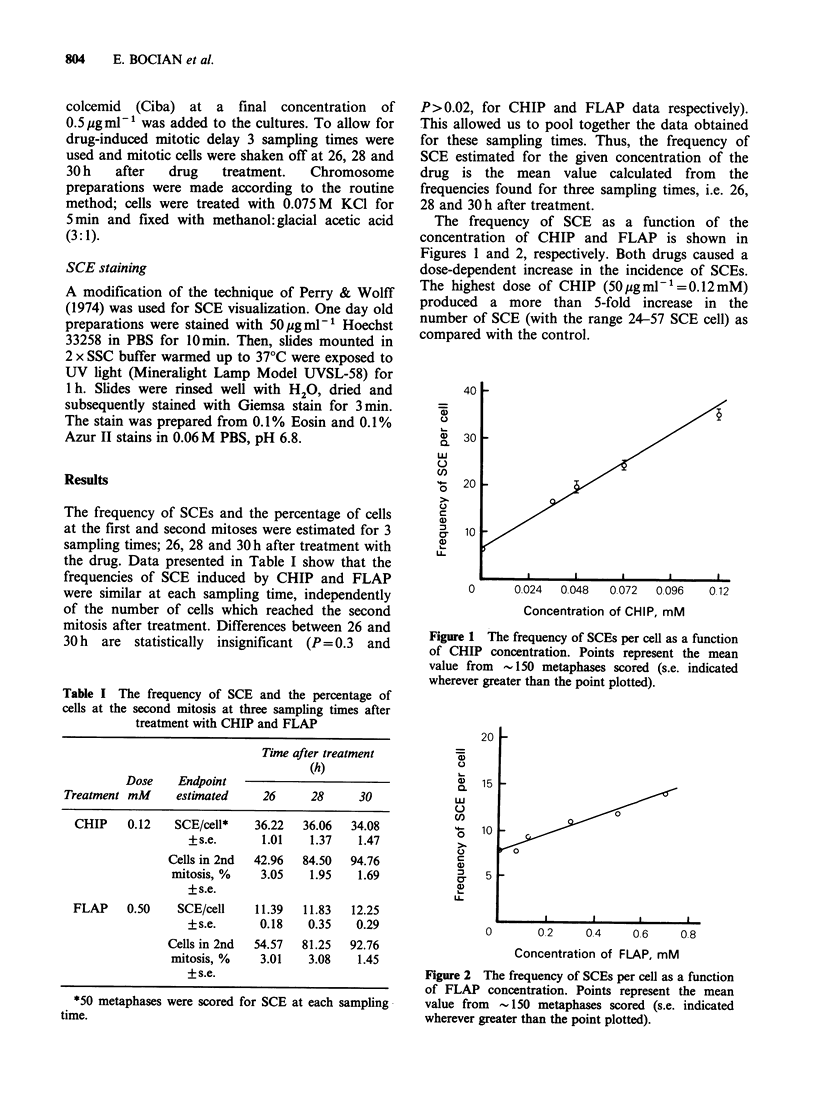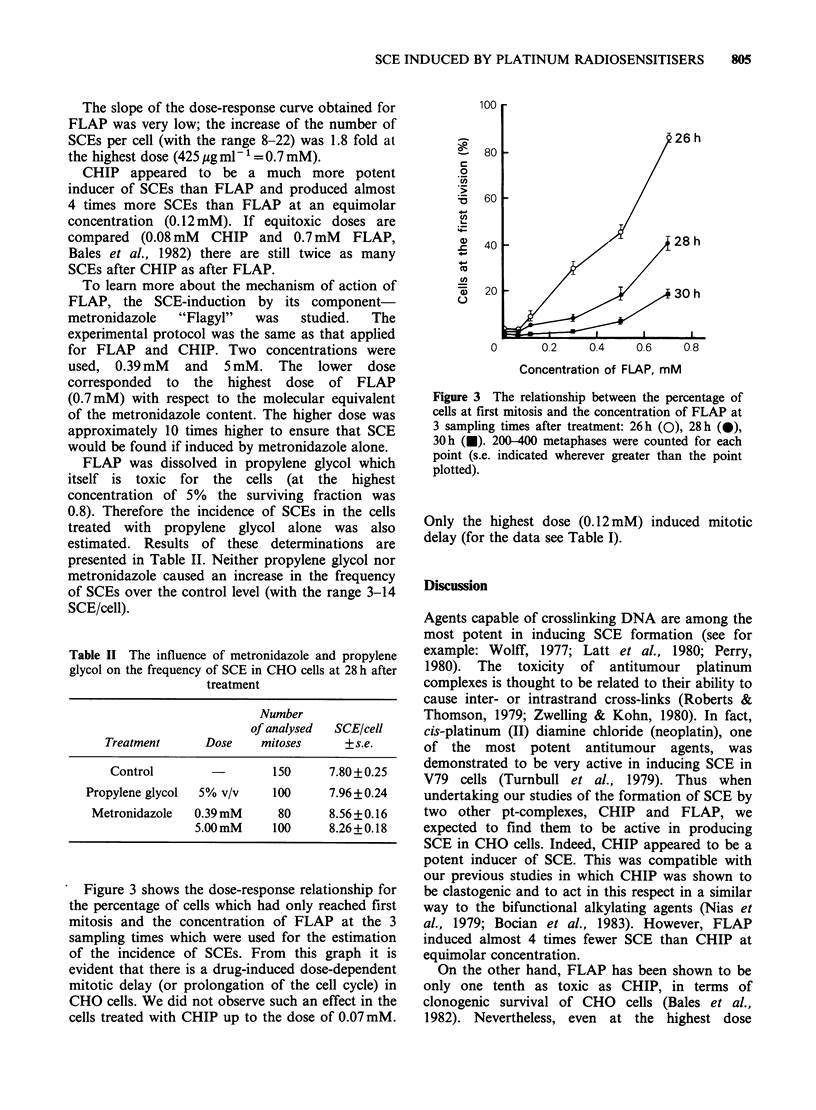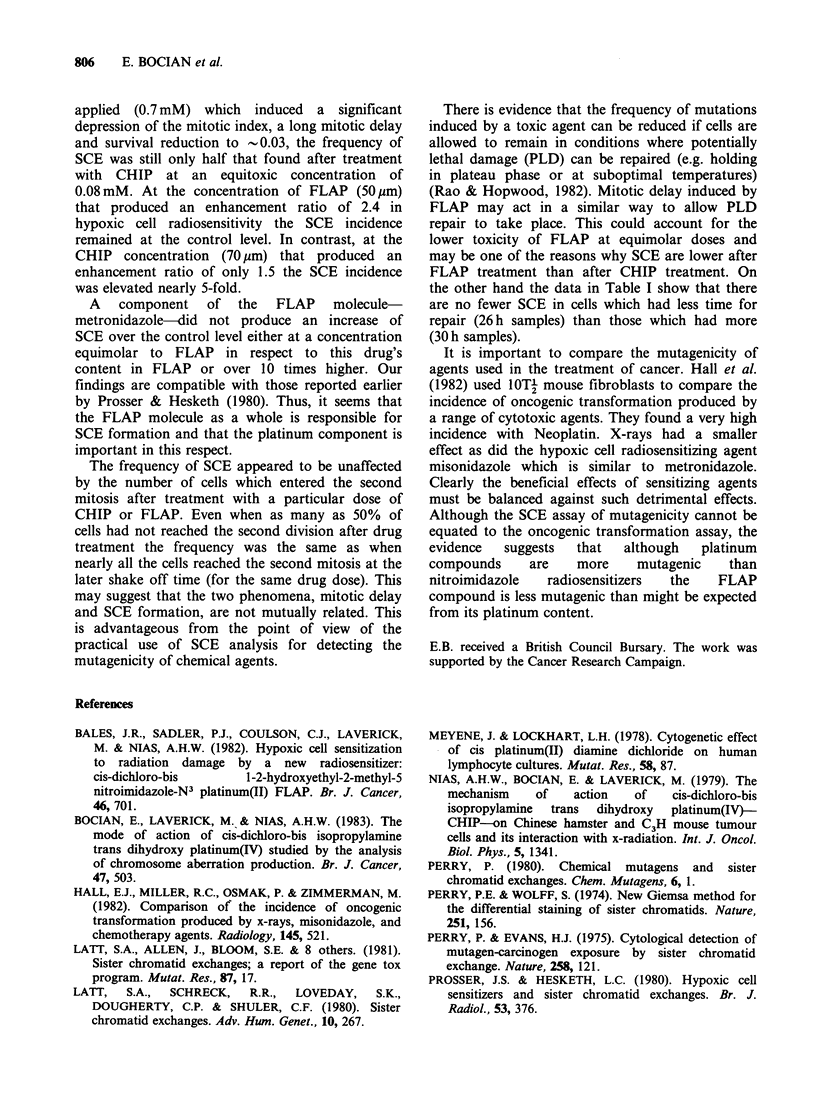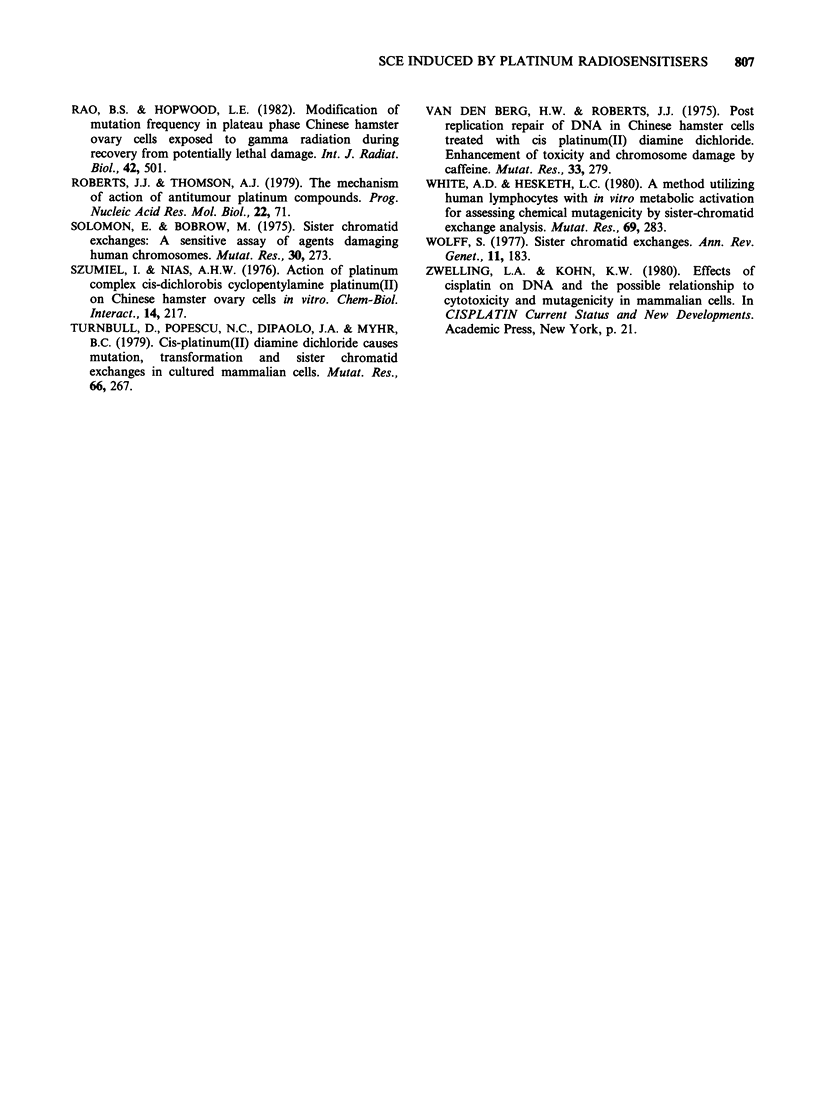# Sister chromatid exchanges induced by two radiosensitizing platinum compounds (cis-dichloro-bis isopropylamine trans dihydroxy platinum IV (CHIP) and cis platinum metronidazole2Cl2(FLAP] in CHO cells in vitro.

**DOI:** 10.1038/bjc.1983.270

**Published:** 1983-12

**Authors:** E. Bocian, M. Laverick, A. H. Nias

## Abstract

Sister chromatid exchange (SCE) induction by two radiosensitizing platinum compounds (cis-dichloro-bis isopropylamine trans dihydroxy platinum IV (CHIP) and cis-platinum metronidazole2 Cl2 (FLAP] was studied in CHO cells in vitro. Both drugs induced SCE in a dose dependent manner. CHIP was a much more potent inducer of SCE than FLAP and produced almost 4 times as many SCE as FLAP at equimolar concentrations and twice as many at equitoxic dosage. Induction of SCE by a component of the FLAP molecule--metronidazole--was also examined. It did not cause any increase of SCE frequency over the control level when applied at 10 times the highest concentration of FLAP which was used.


Br. J. Cancer (1983), **48**, 803–807

### Sister chromatid exchanges induced by two radiosensitizing platinum compounds (*cis*-dichloro-bis isopropylamine trans dihydroxy platinum IV (CHIP) and *cis* platinum metronidazole$_2$Cl$_2$(FLAP)) in CHO cells *in vitro*

E. Bocian[1], M. Laverick[2] & A.H.W. Nias[2]

[1]*Department of Radiobiology and Health Protection, Institute of Nuclear Research, Dorodna 16, PL 03–195 Warszawa, Poland and* [2]*Richard Dimbleby Department of Cancer Research, St. Thomas's Hospital Medical School, London SE1 7EH.*

**Summary** Sister chromatid exchange (SCE) induction by two radiosensitizing platinum compounds (*cis*-dichloro-bis isopropylamine trans dihydroxy platinum IV (CHIP) and *cis*-platinum metronidazole$_2$ Cl$_2$ (FLAP)) was studied in CHO cells *in vitro*. Both drugs induced SCE in a dose dependent manner. CHIP was a much more potent inducer of SCE than FLAP and produced almost 4 times as many SCE as FLAP at equimolar concentrations and twice as many at equitoxic dosage. Induction of SCE by a component of the FLAP molecule—metronidazole—was also examined. It did not cause any increase of SCE frequency over the control level when applied at 10 times the highest concentration of FLAP which was used.

Sister chromatid exchange (SCE) formation has been proposed as a sensitive and simple method for detecting the mutagenicity of chemical agents (Latt *et al.*, 1981, Perry, 1980; Perry & Evans, 1975; Solomon & Bobrow, 1975; White & Hesketh, 1980). The mode of action of various platinum coordination compounds has been extensively studied since their discovery by Rosenberg (for review see Roberts & Thomson, 1979). Some of the compounds have been reported to induce chromosomal aberrations (Van den Berg & Roberts, 1975; Szumiel & Nias, 1976; Meyene & Lockhart, 1978; Nias *et al.*, 1979; Bocian *et al.*, 1983). A positive dose-response relationship for SCE induction by *cis*-platinum II diamine dichloride has also been found (Turnbull *et al.*, 1979).

In this paper we present the results of studies on SCE induction by two other platinum complexes; *cis*-dichloro-bis isopropylamine trans dihydroxy platinum (IV) (CHIP) and *cis*-platinum metronidazole$_2$ Cl$_2$ (FLAP) in CHO cells. CHIP was found in our previous studies (Nias *et al.*, 1979; Bocian *et al.*, 1983) to act in a similar way to the bifunctional alkylating agents with respect to the production of classic structural chromosomal aberrations. FLAP is a new platinum coordination complex which has been shown to be an effective radiosensitizer toward hypoxic cells *in vitro* (Bales *et al.*, 1982) and its mode of action is now being studied *in vivo*.

Correspondence: A.H.W. Nias

Received 21 April 1983; accepted 16 September 1983.

### Materials and methods

### *Cells*

Experiments were carried out on an aneuploid line of CHO cells with 23 chromosomes (Clone 10). Cells were grown in monolayer culture in HEPES buffered Minimal Essential Medium (MEM) supplemented with 15% calf serum, non essential amino acids and L-glutamine. No antibiotics were used. (For details see Bocian *et al.*, 1983).

### *Drug treatment*

Stock solutions of CHIP (supplied by Johnson Matthey Ltd.) and Metronidazole (Sigma) were prepared in physiological saline. FLAP (supplied by May and Baker Ltd) was dissolved in propylene glycol with warming to 70°C for 5 min. Both solutions were kept in the dark and freshly prepared before each experiment. Experiments were designed according to the protocol suggested by the GENE-TOX Work Group on Sister Chromatid Exchanges (Latt *et al.*, 1981). Asynchronous exponential CHO cell cultures 13–14 h after plating $4 \times 10^5$ cells per T75 flask were exposed to the drugs for 1 h at 37°C. The medium containing drug was then removed and replaced by fresh medium containing 5-bromo-deoxyuridine (BUdR) at a final concentration of $10\,\mu g\,ml^{-1}$.

### *Chromosome preparations*

Cultures were grown in the presence of BUdR for the period of two cell cycles (i.e. for 26–30 h) in darkness. Two hours before mitotic collection,

© The Macmillan Press Ltd., 1983

colcemid (Ciba) at a final concentration of 0.5 $\mu g\,ml^{-1}$ was added to the cultures. To allow for drug-induced mitotic delay 3 sampling times were used and mitotic cells were shaken off at 26, 28 and 30 h after drug treatment. Chromosome preparations were made according to the routine method; cells were treated with 0.075 M KCl for 5 min and fixed with methanol: glacial acetic acid (3:1).

*SCE staining*

A modification of the technique of Perry & Wolff (1974) was used for SCE visualization. One day old preparations were stained with 50 $\mu g\,ml^{-1}$ Hoechst 33258 in PBS for 10 min. Then, slides mounted in 2 x SSC buffer warmed up to 37°C were exposed to UV light (Mineralight Lamp Model UVSL-58) for 1 h. Slides were rinsed well with $H_2O$, dried and subsequently stained with Giemsa stain for 3 min. The stain was prepared from 0.1% Eosin and 0.1% Azur II stains in 0.06 M PBS, pH 6.8.

### Results

The frequency of SCEs and the percentage of cells at the first and second mitoses were estimated for 3 sampling times; 26, 28 and 30 h after treatment with the drug. Data presented in Table I show that the frequencies of SCE induced by CHIP and FLAP were similar at each sampling time, independently of the number of cells which reached the second mitosis after treatment. Differences between 26 and 30 h are statistically insignificant ($P=0.3$ and $P>0.02$, for CHIP and FLAP data respectively). This allowed us to pool together the data obtained for these sampling times. Thus, the frequency of SCE estimated for the given concentration of the drug is the mean value calculated from the frequencies found for three sampling times, i.e. 26, 28 and 30 h after treatment.

The frequency of SCE as a function of the concentration of CHIP and FLAP is shown in Figures 1 and 2, respectively. Both drugs caused a dose-dependent increase in the incidence of SCEs. The highest dose of CHIP (50 $\mu g\,ml^{-1}=0.12$ mM) produced a more than 5-fold increase in the number of SCE (with the range 24–57 SCE cell) as compared with the control.

**Table I** The frequency of SCE and the percentage of cells at the second mitosis at three sampling times after treatment with CHIP and FLAP

| Treatment | Dose mM | Endpoint estimated | Time after treatment (h) 26 | 28 | 30 |
|---|---|---|---|---|---|
| CHIP | 0.12 | SCE/cell* | 36.22 | 36.06 | 34.08 |
| | | ±s.e. | 1.01 | 1.37 | 1.47 |
| | | Cells in 2nd mitosis, % | 42.96 | 84.50 | 94.76 |
| | | ±s.e. | 3.05 | 1.95 | 1.69 |
| FLAP | 0.50 | SCE/cell | 11.39 | 11.83 | 12.25 |
| | | ±s.e. | 0.18 | 0.35 | 0.29 |
| | | Cells in 2nd mitosis, % | 54.57 | 81.25 | 92.76 |
| | | ±s.e. | 3.01 | 3.08 | 1.45 |

*50 metaphases were scored for SCE at each sampling time.

**Figure 1** The frequency of SCEs per cell as a function of CHIP concentration. Points represent the mean value from ~150 metaphases scored (s.e. indicated wherever greater than the point plotted).

**Figure 2** The frequency of SCEs per cell as a function of FLAP concentration. Points represent the mean value from ~150 metaphases scored (s.e. indicated wherever greater than the point plotted).

The slope of the dose-response curve obtained for FLAP was very low; the increase of the number of SCEs per cell (with the range 8–22) was 1.8 fold at the highest dose (425 $\mu g\,ml^{-1} = 0.7\,mM$).

CHIP appeared to be a much more potent inducer of SCEs than FLAP and produced almost 4 times more SCEs than FLAP at an equimolar concentration (0.12 mM). If equitoxic doses are compared (0.08 mM CHIP and 0.7 mM FLAP, Bales *et al.*, 1982) there are still twice as many SCEs after CHIP as after FLAP.

To learn more about the mechanism of action of FLAP, the SCE-induction by its component—metronidazole "Flagyl" was studied. The experimental protocol was the same as that applied for FLAP and CHIP. Two concentrations were used, 0.39 mM and 5 mM. The lower dose corresponded to the highest dose of FLAP (0.7 mM) with respect to the molecular equivalent of the metronidazole content. The higher dose was approximately 10 times higher to ensure that SCE would be found if induced by metronidazole alone.

FLAP was dissolved in propylene glycol which itself is toxic for the cells (at the highest concentration of 5% the surviving fraction was 0.8). Therefore the incidence of SCEs in the cells treated with propylene glycol alone was also estimated. Results of these determinations are presented in Table II. Neither propylene glycol nor metronidazole caused an increase in the frequency of SCEs over the control level (with the range 3–14 SCE/cell).

**Table II** The influence of metronidazole and propylene glycol on the frequency of SCE in CHO cells at 28 h after treatment

| *Treatment* | *Dose* | *Number of analysed mitoses* | *SCE/cell ±s.e.* |
|---|---|---|---|
| Control | — | 150 | 7.80±0.25 |
| Propylene glycol | 5% v/v | 100 | 7.96±0.24 |
| Metronidazole | 0.39 mM | 80 | 8.56±0.16 |
| | 5.00 mM | 100 | 8.26±0.18 |

Figure 3 shows the dose-response relationship for the percentage of cells which had only reached first mitosis and the concentration of FLAP at the 3 sampling times which were used for the estimation of the incidence of SCEs. From this graph it is evident that there is a drug-induced dose-dependent mitotic delay (or prolongation of the cell cycle) in CHO cells. We did not observe such an effect in the cells treated with CHIP up to the dose of 0.07 mM.

**Figure 3** The relationship between the percentage of cells at first mitosis and the concentration of FLAP at 3 sampling times after treatment: 26 h (○), 28 h (●), 30 h (■). 200–400 metaphases were counted for each point (s.e. indicated wherever greater than the point plotted).

Only the highest dose (0.12 mM) induced mitotic delay (for the data see Table I).

### Discussion

Agents capable of crosslinking DNA are among the most potent in inducing SCE formation (see for example: Wolff, 1977; Latt *et al.*, 1980; Perry, 1980). The toxicity of antitumour platinum complexes is thought to be related to their ability to cause inter- or intrastrand cross-links (Roberts & Thomson, 1979; Zwelling & Kohn, 1980). In fact, *cis*-platinum (II) diamine chloride (neoplatin), one of the most potent antitumour agents, was demonstrated to be very active in inducing SCE in V79 cells (Turnbull *et al.*, 1979). Thus when undertaking our studies of the formation of SCE by two other pt-complexes, CHIP and FLAP, we expected to find them to be active in producing SCE in CHO cells. Indeed, CHIP appeared to be a potent inducer of SCE. This was compatible with our previous studies in which CHIP was shown to be clastogenic and to act in this respect in a similar way to the bifunctional alkylating agents (Nias *et al.*, 1979; Bocian *et al.*, 1983). However, FLAP induced almost 4 times fewer SCE than CHIP at equimolar concentration.

On the other hand, FLAP has been shown to be only one tenth as toxic as CHIP, in terms of clonogenic survival of CHO cells (Bales *et al.*, 1982). Nevertheless, even at the highest dose applied (0.7 mM) which induced a significant depression of the mitotic index, a long mitotic delay and survival reduction to ~0.03, the frequency of SCE was still only half that found after treatment with CHIP at an equitoxic concentration of 0.08 mM. At the concentration of FLAP (50 $\mu$m) that produced an enhancement ratio of 2.4 in hypoxic cell radiosensitivity the SCE incidence remained at the control level. In contrast, at the CHIP concentration (70 $\mu$m) that produced an enhancement ratio of only 1.5 the SCE incidence was elevated nearly 5-fold.

A component of the FLAP molecule—metronidazole—did not produce an increase of SCE over the control level either at a concentration equimolar to FLAP in respect to this drug's content in FLAP or over 10 times higher. Our findings are compatible with those reported earlier by Prosser & Hesketh (1980). Thus, it seems that the FLAP molecule as a whole is responsible for SCE formation and that the platinum component is important in this respect.

The frequency of SCE appeared to be unaffected by the number of cells which entered the second mitosis after treatment with a particular dose of CHIP or FLAP. Even when as many as 50% of cells had not reached the second division after drug treatment the frequency was the same as when nearly all the cells reached the second mitosis at the later shake off time (for the same drug dose). This may suggest that the two phenomena, mitotic delay and SCE formation, are not mutually related. This is advantageous from the point of view of the practical use of SCE analysis for detecting the mutagenicity of chemical agents.

There is evidence that the frequency of mutations induced by a toxic agent can be reduced if cells are allowed to remain in conditions where potentially lethal damage (PLD) can be repaired (e.g. holding in plateau phase or at suboptimal temperatures) (Rao & Hopwood, 1982). Mitotic delay induced by FLAP may act in a similar way to allow PLD repair to take place. This could account for the lower toxicity of FLAP at equimolar doses and may be one of the reasons why SCE are lower after FLAP treatment than after CHIP treatment. On the other hand the data in Table I show that there are no fewer SCE in cells which had less time for repair (26 h samples) than those which had more (30 h samples).

It is important to compare the mutagenicity of agents used in the treatment of cancer. Hall *et al.* (1982) used 10T$\frac{1}{2}$ mouse fibroblasts to compare the incidence of oncogenic transformation produced by a range of cytotoxic agents. They found a very high incidence with Neoplatin. X-rays had a smaller effect as did the hypoxic cell radiosensitizing agent misonidazole which is similar to metronidazole. Clearly the beneficial effects of sensitizing agents must be balanced against such detrimental effects. Although the SCE assay of mutagenicity cannot be equated to the oncogenic transformation assay, the evidence suggests that although platinum compounds are more mutagenic than nitroimidazole radiosensitizers the FLAP compound is less mutagenic than might be expected from its platinum content.

E.B. received a British Council Bursary. The work was supported by the Cancer Research Campaign.

### References

BALES, J.R., SADLER, P.J., COULSON, C.J., LAVERICK, M. & NIAS, A.H.W. (1982). Hypoxic cell sensitization to radiation damage by a new radiosensitizer: cis-dichloro-bis 1-2-hydroxyethyl-2-methyl-5 nitroimidazole-N[3] platinum(II) FLAP. *Br. J. Cancer*, **46**, 701.

BOCIAN, E., LAVERICK, M. & NIAS, A.H.W. (1983). The mode of action of cis-dichloro-bis isopropylamine trans dihydroxy platinum(IV) studied by the analysis of chromosome aberration production. *Br. J. Cancer*, **47**, 503.

HALL, E.J., MILLER, R.C., OSMAK, P. & ZIMMERMAN, M. (1982). Comparison of the incidence of oncogenic transformation produced by x-rays, misonidazole, and chemotherapy agents. *Radiology*, **145**, 521.

LATT, S.A., ALLEN, J., BLOOM, S.E. & 8 others. (1981). Sister chromatid exchanges; a report of the gene tox program. *Mutat. Res.*, **87**, 17.

LATT, S.A., SCHRECK, R.R., LOVEDAY, S.K., DOUGHERTY, C.P. & SHULER, C.F. (1980). Sister chromatid exchanges. *Adv. Hum. Genet.*, **10**, 267.

MEYENE, J. & LOCKHART, L.H. (1978). Cytogenetic effect of cis platinum(II) diamine dichloride on human lymphocyte cultures. *Mutat. Res.*, **58**, 87.

NIAS, A.H.W., BOCIAN, E. & LAVERICK, M. (1979). The mechanism of action of cis-dichloro-bis isopropylamine trans dihydroxy platinum(IV)—CHIP—on Chinese hamster and $C_3H$ mouse tumour cells and its interaction with x-radiation. *Int. J. Oncol. Biol. Phys.*, **5**, 1341.

PERRY, P. (1980). Chemical mutagens and sister chromatid exchanges. *Chem. Mutagens*, **6**, 1.

PERRY, P.E. & WOLFF, S. (1974). New Giemsa method for the differential staining of sister chromatids. *Nature*, **251**, 156.

PERRY, P. & EVANS, H.J. (1975). Cytological detection of mutagen-carcinogen exposure by sister chromatid exchange. *Nature*, **258**, 121.

PROSSER, J.S. & HESKETH, L.C. (1980). Hypoxic cell sensitizers and sister chromatid exchanges. *Br. J. Radiol.*, **53**, 376.

RAO, B.S. & HOPWOOD, L.E. (1982). Modification of mutation frequency in plateau phase Chinese hamster ovary cells exposed to gamma radiation during recovery from potentially lethal damage. *Int. J. Radiat. Biol.*, **42**, 501.

ROBERTS, J.J. & THOMSON, A.J. (1979). The mechanism of action of antitumour platinum compounds. *Prog. Nucleic Acid Res. Mol. Biol.*, **22**, 71.

SOLOMON, E. & BOBROW, M. (1975). Sister chromatid exchanges: A sensitive assay of agents damaging human chromosomes. *Mutat. Res.*, **30**, 273.

SZUMIEL, I. & NIAS, A.H.W. (1976). Action of platinum complex cis-dichlorobis cyclopentylamine platinum(II) on Chinese hamster ovary cells *in vitro*. *Chem-Biol. Interact.*, **14**, 217.

TURNBULL, D., POPESCU, N.C., DIPAOLO, J.A. & MYHR, B.C. (1979). Cis-platinum(II) diamine dichloride causes mutation, transformation and sister chromatid exchanges in cultured mammalian cells. *Mutat. Res.*, **66**, 267.

VAN DEN BERG, H.W. & ROBERTS, J.J. (1975). Post replication repair of DNA in Chinese hamster cells treated with cis platinum(II) diamine dichloride. Enhancement of toxicity and chromosome damage by caffeine. *Mutat. Res.*, **33**, 279.

WHITE, A.D. & HESKETH, L.C. (1980). A method utilizing human lymphocytes with *in vitro* metabolic activation for assessing chemical mutagenicity by sister-chromatid exchange analysis. *Mutat. Res.*, **69**, 283.

WOLFF, S. (1977). Sister chromatid exchanges. *Ann. Rev. Genet.*, **11**, 183.

ZWELLING, L.A. & KOHN, K.W. (1980). Effects of cisplatin on DNA and the possible relationship to cytotoxicity and mutagenicity in mammalian cells. In *CISPLATIN Current Status and New Developments*. Academic Press, New York, p. 21.